# The core protein of a pestivirus protects the incoming virus against IFN-induced effectors

**DOI:** 10.1038/srep44459

**Published:** 2017-03-14

**Authors:** Christiane Riedel, Benjamin Lamp, Benedikt Hagen, Stanislav Indik, Till Rümenapf

**Affiliations:** 1 Institute of Virology, Vetmeduni Vienna, Veterinärplatz 1, 1210 Vienna, Austria

## Abstract

A multitude of viral factors - either inhibiting the induction of the IFN-system or its effectors – have been described to date. However, little is known about the role of structural components of the incoming virus particle in protecting against IFN-induced antiviral factors during or immediately after entry. In this study, we take advantage of the previously reported property of *Classical swine fever virus* (family *Flaviviridae*, genus *Pestivirus*) to tolerate a deletion of the core protein if a compensatory mutation is present in the NS3-helicase-domain (Vp447_∆c_). In contrast to the parental virus (Vp447), which causes a hemorrhagic-fever-like disease in pigs, Vp447_∆c_ is avirulent *in vivo*. In comparison to Vp447, growth of Vp447_∆c_ in primary porcine cells and IFN-treated porcine cell lines was reduced >20-fold. Also, primary porcine endothelial cells and IFN-pretreated porcine cell lines were 8–24 times less susceptible to Vp447_∆c_. This reduction of susceptibility could be partially reversed by loading Vp447_∆c_ particles with different levels of core protein. In contrast, expression of core protein in the recipient cell did not have any beneficial effect. Therefore, a protective effect of core protein in the incoming virus particle against the products of IFN-stimulated genes could be demonstrated.

The IFN-system is a powerful cellular tool to counteract virus infection at different levels (as reviewed in refs 1, 2, 3, 4). Activation of the IFN-system either occurs by IFNs present in the medium surrounding the cells or by contact of cellular pattern recognition receptors with pathogen associated molecular patterns. Subsequently, a complex messenger cascade leads – amongst others – to the expression of IFN-stimulated genes (ISGs). Known antiviral effects of ISGs are for example degradation of RNA (RNaseL/ISG20), inhibition of protein synthesis (PKR) and RNA mismatching (ADAR).

*Pestivirus* is a genus within the family *Flaviviridae* that contains pathogens of cloven-hooved animals. *Classical swine fever virus* (CSFV) and *Bovine viral diarrhea virus* (BVDV) can be responsible for substantial economic losses and are notifiable in several countries. All pestivirus species can be transmitted vertically, and depending on the time point of infection of the fetuses, the results are fetal death, malformations or – if infection takes place before the development of the active immune response - the birth of persistently infected, often phenotypically normal offspring, which is immunotolerant to the virus^5^ as no adaptive immunity is mounted. This is of pivotal epidemiological importance, but also implies that the virus is able to selectively evade the innate immune response of its host, as the virus is replicating within these persistently infected animals without causing immunosuppression.

Two proteins unique to pestiviruses – Npro and E^rns^ – are known inhibitors of induction of the innate immune response. Npro, the N-terminal autoprotease^6^, is inducing the proteasomal degradation of IFN regulatory factor 3 (IRF3)^7^,^8^,^9^. On the other hand, E^rns^, a surface glycoprotein with RNase activity^10^,^11^, inhibits activation of the IFN-system by extracellular (pestiviral) RNA^12^,^13^,^14^ and sensing of infected cells by plasmacytoid dendritic cells^15^.

However, high IFN-α levels can be detected in CSFV infected pigs^16^, most likely originating from the usage of IRF7 instead of IRF3 in plasmacytoid dendritic cells^17^. Furthermore, evidence exists that IFN-induced genes are upregulated in persistently BVDV infected animals^18^,^19^,^20^,^21^. Yet, no factors protecting the virus against effectors of the IFN-system – apart from the utilization of IRES mediated translation initiation, which should render the virus independent of certain elongation initiation factors (eIF1A+B, 4A,4B,4F)^22^ and less sensitive to PKR mediated phosphorylation of eIF2alpha^23^ - have been discovered to date.

Pestivirus particles contain four known structural proteinaceous components (reviewed in ref. 24). The glycoproteins E^rns^, E1 and E2 are located within the viral envelope and interact with the target cell. Inside the virus particle, core protein is associated with the viral genome, supposedly forming a nucleocapsid structure. However, high resolution cryo electron microscopy reconstructions of virus particles from several members of the *Flaviviridae* – including Dengue and Zika virus^25^,^26^ - did not provide any evidence for the existence of a highly ordered nucleocapsid. Also, the mechanism of RNA genome encapsidation is still poorly understood. The discovery of Pegiviruses within the *Flaviviridae* - with no apparent core coding region in several members^27^ – implies that core independent mechanisms of RNA encapsidation and particle generation exist. With the finding that a single amino acid substitution within the nonstructural protein 3 (NS3) helicase domain can compensate for loss of the core coding region in CSFV^28^, also providing evidence for yet unknown functions of core protein. The only phenotypic differences between unmodified CSFV (Vp447) and the core deletion mutant (Vp447_∆c_) were a ± 10-fold drop in virus titer *in vitro* and avirulence in the natural host. The aim of this study was to uncover additional functions of core protein by focusing on the potential reason(s) for its attenuation *in vivo*.

## Results

### Vp447_∆c_ is more sensitive to an activated IFN-system

**Figure 1:**
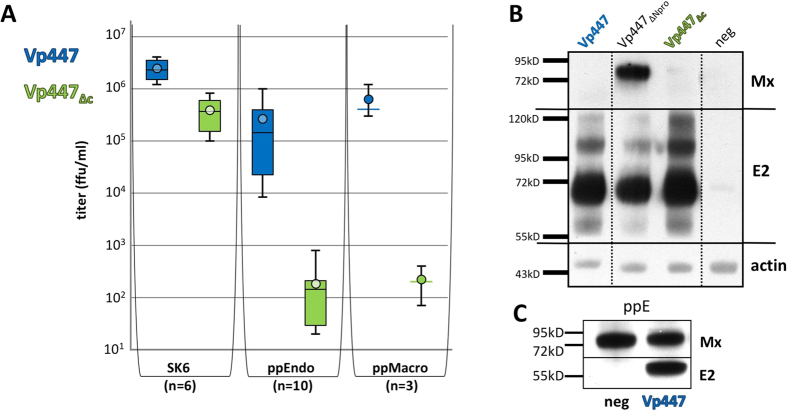
Growth of Vp447 and Vp447_∆c_ in primary porcine cells and SK6-cells (**A**), their capacity to suppress induction of MX1 expression in PK15-cells (**B**) and expression of MX1 in ppE (**C**). (**A**) Titer of Vp447 and Vp447_∆c_ 48 h after infection of SK6-cells (n = 6), ppEndo (n = 10) and ppMacro (n = 3). Depicted are median, minimum, maximum and average values (circle), as well as the quartiles if applicable. (**B**) Detection of MX1, E2 and β-actin by Western Blot analysis in lysates of PK15-cells either infected with Vp447, Vp447_∆c_, a Vp447 Npro deletion mutant (Vp447_∆Npro_) or mock infected (neg). (**C**) Detection of MX1 and E2 by Western blot analysis in lysates of Vp447 infected or mock infected ppEndo (neg).

*In vitro*, Vp447_∆c_ did not exhibit an overt phenotype when compared to the parental virus except for an approximately 10-fold reduced average virus titer 48 h after infection (SK6-cells: 8-fold reduction; PK15-cells: 14-fold reduction and STE-cells: 9-fold reduction; n = 6) (displayed in Fig. 1A for SK6-cells). To address potential causes for the strong attenuation of Vp447_∆c_ *in vivo*, the growth of Vp447_∆c_ was tested in primary porcine cells. 48 h after infection of primary porcine aortic endothelial cells (ppEndo) and primary porcine macrophages (ppMacro, derived from PBMCs) with the parental Vp447, on average 2.7 × 10^5^ (ppEndo) and 6.3 × 10^5^ ffu/ml (ppMacro), respectively, could be detected in the supernatant (Fig. 1A). When compared to SK6-cells this represents a 10- and 4-fold reduction, respectively. Infection with Vp447_∆c_ resulted in an average titer of 1.8 × 10^2^ (ppEndo) and 2.2 × 10^2^ ffu/ml (ppMacro) after 48 h (Fig. 1A). This translates into a 2000-fold reduction when compared to SK6-cells. In comparison to standard porcine cell lines the utilization of primary cells allowed to reveal a more pronounced phenotype of Vp447_∆c_ that is characterized by a more than 10^3^ –fold reduced titer compared to Vp447.

The observed properties of Vp447_∆c_ resembled the phenotype of CSFV mutants in which the Npro gene had been deleted^29^,^30^. To analyze whether the more pronounced titer reduction of Vp447_∆c_ in primary cells might be correlated to the activation of the IFN-system, the expression of the strictly IFN-induced MX1 protein was assessed^31^. Infection of PK15-cells with Vp447_∆c_ or with Vp447 did not induce MX1 expression, but the protein was easily detectable upon infection with a Vp447 Npro deletion mutant (Fig. 1B). This result suggests that the lack of core protein does not compromise the function of Npro as antagonist of IRF3. During infection experiments with ppEndo cells we observed strong MX1 expression not only after infection with Vp447 but also in the non-infected control (Fig. 1C). The underlying cause of this activation is unknown. Yet, this unexpected finding raised the question whether activation of the innate immune system was – at least partially - responsible for the meagre growth of Vp447_∆c_ in ppEndo. To test this hypothesis PK15-cells were treated with human IFN-α either 16 h prior or 4 h after infection with Vp447 or Vp447_∆c_. 48 h after infection, Vp447 titers were reduced less than 10-fold irrespective of the time point of IFN-treatment when compared to the untreated control. In contrast, Vp447_∆c_ titers were reduced more than 50-fold if cells were treated with IFN 4 h after infection, and more than 200-fold when cells were treated 16 h before infection (n = 3, data not shown).

**Figure 2:**
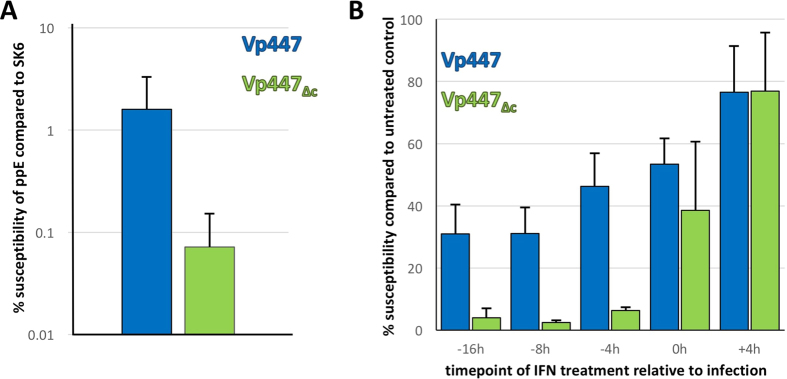
Relative susceptibility of ppEndo (**A**) and IFN-pretreated PK15-cells (**B**) to Vp447_∆c_ is reduced. (**A**) Relative susceptibility to Vp447 and Vp447_∆c_ in percent was calculated as fraction of infected ppE/infected SK6-cells (n = 8). (**B**) PK15-cells were treated with IFN-α 16, 8 or 4 hours before infection (−16 h, −8 h, −4 h), at the timepoint of infection (0 h) and 4 hours after infection (+4 h) (n = 3). Susceptibility to Vp447 and Vp447_∆c_ in percent was calculated as fraction of infected IFN-treated PK15-cells/infected mock treated PK15-cells. Displayed are mean and standard deviation.

Virus titer as sole readout for cellular effects on virus propagation is rather unprecise as it cannot distinguish the different stages of a virus’ lifecycle (e.g. entry, replication, release). In an attempt to reduce the affected stages to entry and (early) replication, we determined the number of infected ppEndo and the number of infected SK6-cells for the same amount of input virus. These data were then used to calculate the relative susceptibility of ppEndo (in %) when compared to SK6-cells as a measure for the increased resistance of ppEndo to infection by Vp447 and Vp447_∆c_. The relative susceptibility of ppEndo for Vp447 was 1.6% and 0.07% for Vp447_∆c_. This represents a 22-fold reduction of the susceptibility of ppEndo for Vp447_∆c_ in comparison to Vp447 (Fig. 2A).

The same assay was used to assess the effect of IFN-α treatment at different time points (16, 8, 4 hours before infection, at infection and 4 hours after infection) on the susceptibility of PK15-cells (Fig. 2B). Here, relative susceptibility was calculated as percentage of infected IFN-treated PK15-cells compared to infected mock-treated PK15-cells. The effect of IFN-pretreatment was mild for Vp447, with average susceptibilities ranging from 31% (16 h before infection) to 77% (4 h after infection). Susceptibility to Vp447_∆c_ ranged from 3.9% (16 h before infection) to 77% (4 h after infection), demonstrating an increased sensitivity of Vp447_∆c_ to IFN-pretreatment of host cells. Similar results were obtained when STE-cells were treated with IFN-α 16 h before infection, with average 16.4% susceptibility for Vp447 and 0.69% for Vp447_∆c_ (n = 3). Collectively, these data suggest that the ability of Vp447_∆c_ to infect cells with an activated IFN-system is markedly reduced in comparison to Vp447.

#### The amount of core protein in the virion determines its infectivity for IFN-treated cells

The more pronounced effect of IFN-treatment on the susceptibility of Vp447_∆c_ can be taken as evidence for a protective role of core protein during either entry or replication. To elucidate whether the replication of Vp447 and Vp447_∆c_ genomes was differently affected by IFN-treatment, IFN-treated PK15-cells as well untreated SK6- and PK15-cells were transfected with standardized amounts of Vp447 and Vp447_∆c_ genomic RNA. The average specific infectivity per μg of genome of Vp447 was 2.4 × 10^5^ SK6-cells and 1.5 × 10^5^ PK15-cells, respectively. Comparable values were observed for Vp447_∆c_ genomes, with 3.5 × 10^5^ antigen positive cells/μg for SK6-cells and 2.8 × 10^5^ for PK15-cells. Transfection of IFN-pretreated cells led to a 13- and 22-fold drop of specific infectivity for Vp447 and Vp447_∆c_ genomes, respectively. This translates into a less than 2-fold reduced specific infectivity of Vp447_∆c_ genomes when compared to Vp447, which makes it unlikely that newly produced core protein is a major factor underlying the observed loss of infectivity of Vp447_∆c_ when the cellular IFN-system is activated.

**Figure 3:**
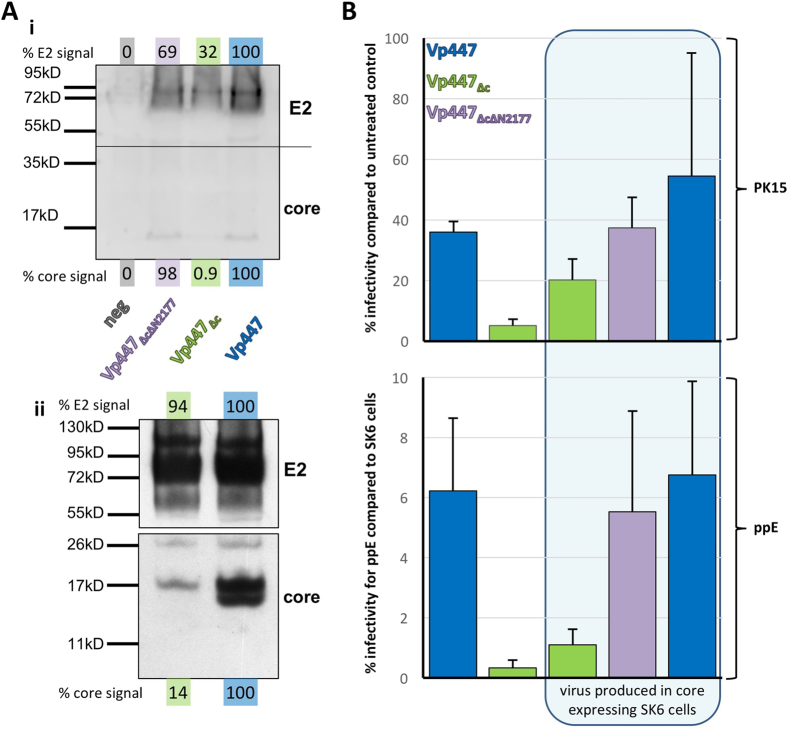
Effect of core transcomplementation on infectivity of Vp447_∆c_. (Ai) Western blot analysis to detect E2 and core in pelleted Vp447_∆cN2177_, Vp447_∆c_ and Vp447 particles produced in core expressing SK6-cells.(Aii) Presence of core in Vp447_∆c_ can be detected after long exposure but is considerably reduced in comparison to Vp447. Signal in (Ai) was quantified employing a C-DiGit Scanner (Licor), whereas signal in (Aii) was quantified in ImageJ^32^ from scanned film. E2 and core signals of Vp447 were defined as 100% and signal in the negative control lane as 0% as no specific band could be detected. (**B**) Relative infectivity of Vp447_∆cN2177_, Vp447_∆c_ and Vp447 produced in SK6-cells or SK6-cells expressing core protein when employing PK15-cells treated with IFN-α 16 h before infection (n = 5) or ppEndo (ppE) (n = 7). Depicted are mean and standard deviation.

Therefore, loading Vp447_∆c_ particles with core protein in trans could have a more pronounced effect regarding the ability to establish an infection in cells with an activated IFN-system. To this end a cell line expressing core protein from a tetracycline-inducible promoter was established. Overexpressed core protein is not efficiently incorporated into Vp447_∆c_ particles due to an antagonistic effect of the rescue mutation Tyr_2177_ in the helicase domain of NS3^28^. To be able to load Vp447_∆c_ particles with amounts of core protein comparable to the parental virus, Vp447_∆c_ lacking the N2177Y rescue mutation (Vp447_∆cN2177_) was also included in the experiments. Vp447_∆cN2177_ can be rescued if core protein is provided in trans. Core protein was readily detectable by Western blot analysis of concentrated Vp447 and Vp447_∆cN2177_ particles (Fig. 3Ai). Interestingly, the ratio of core:E2 signal was on average elevated two-fold (n = 3) for Vp447_∆cN2177_ in comparison to Vp447. A weak core band could only be detected in Vp447_∆c_ particles after prolonged exposure (Fig. 3Aii), resulting in a nearly 7-fold reduction of the core:E2 signal ratio. The origin of the core double band within Vp447 particles is unclear, but has been previously described in highly purified and concentrated BVDV particles^33^.

Subsequently, Vp447, Vp447_∆c_ and Vp447_∆cN2177_ were produced in core-expressing or control cells. This should provide viruses containing different amounts of core protein – if core was provided in trans by the producer cell – or no core protein in the case of Vp447_∆c_ produced in control cells. As the following experiments aim at elucidating properties of the virus and not of the cell, the term infectivity will replace susceptibility. In the following experiment infectivity of viruses containing different amounts of core protein was tested employing PK15-cells treated with IFN-α 16 h before infection. A 4-fold increase of infectivity of core-transcomplemented Vp447_∆c_ compared to core protein-lacking Vp447_∆c_ was observed. In the same experimental setup, infectivity of core-transcomplemented Vp447_∆cN2177_ reached levels comparable to the parental virus (Fig. 3B). For STE-cells treated with IFN-α 16 h before infection, average infectivity increased from 0.8% to 10% for core–transcomplemented Vp447_∆c_ and to 13% for Vp447_∆cN2177_, respectively (n = 3, data not shown). Hence, infectivity of core-transcomplemented Vp447_∆cN2177_ also nearly reached levels observed for Vp447 (16%).

To examine the above described effect also in primary porcine cells, the change of infectivity of Vp447_∆c_ and Vp447_∆cN2177_ upon core-transcomplementation was assessed employing ppEndo. In this model, infectivity of core-transcomplemented viruses increased from 0.33% to 1.1 for Vp447_∆c_ and reached 5.5% for Vp447_∆cN2177_. The infection rate observed for the core-loaded Vp447_∆cN2177_ again approached levels observed for Vp447 (6.2%). From these data, it can be concluded that core protein antagonizes IFN-induced effectors that prevent infection of IFN-treated cells.

**Figure 4:**
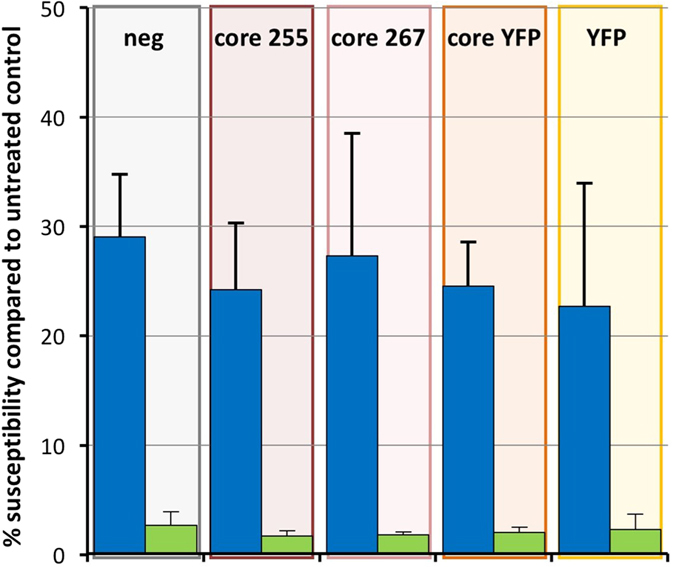
Core expression within PK15-cells does not affect their susceptibility for Vp447_∆c_. Susceptibility of different PK15-derived cell lines expressing core protein (core 255 = core coding sequence up to signal peptide peptidase cleavage site; core 267 = full length core coding sequence; core YFP = full core coding sequence + C-terminal YFP) or YFP for Vp447 and Vp447_∆c_ after IFN-treatment 16 h before infection compared to mock treated control (n = 3). Depicted are mean and standard deviation.

To exclude any effects of core protein expression within the target cell on its susceptibility, PK15-derived cell lines constitutively expressing several core protein variants differing in the length of the C-terminal domain (core 255, core 267, core 267 YFP [numbers represent C-terminal amino acid in Vp447]) and YFP as a control were generated using lentiviral transduction. Different core protein C-termini were chosen to account for its potential effects on subcellular localization/processing of core protein. Upon infection of these cell lines with Vp447_∆c_, no beneficial effect compared to the controls was observed when cells were treated with IFN-α 16 h before infection, as susceptibility ranged between 1.7–2.7% (Fig. 4). This was also the case for the parental virus, Vp447, whose susceptibility ranged between 23 and 29%. Therefore, a protective role of core protein as an integral part of the virus particle against IFN-induced effectors is likely.

## Discussion

The ability of pestiviruses to efficiently modify the host innate immune system has been the center of intense research efforts during the past decades. By now, the functions of Npro and E^rns^ are well defined regarding their abilities to prevent activation of the IFN-system. Yet, IFN- levels are often high in CSFV infected pigs or persistently BVDV infected animals^16^,^17^,^19^,^20^,^21^,^32^,^34^. This implies the existence of viral factors able to counteract effectors of the IFN-system^35^. Until now, little is known about IFN-effectors that can inhibit pestivirus growth and the viral components that allow pestiviruses to stay unaffected by a cellular defense state that efficiently inhibits another virus upon coinfection.

The construction of a CSFV that tolerated a nearly complete deletion of the core protein allowed for the first time to examine the effect of a missing structural virus component on its growth *in vitro* and *in vivo*. The previously reported animal experiment demonstrated the need for core protein in the natural host^28^. However, this did not reveal any specific function of core protein and the observed behavior *in vivo* could either be due to a specific effect of core protein, or just be invoked by the less efficient virus production of Vp447_∆c_ observed *in vitro*.

The reduced infectivity of Vp447_∆c_ for primary cells and IFN-activated cell lines indicated that core protein is fulfilling (an) essential role(s) during the viral life cycle. When looking at the far better characterized HCV core protein, multiple interactions with cellular components have been described, including modification of the innate immune response by negatively regulating the JAK-STAT pathway (reviewed in ref. 36). However, pestiviruses have been shown not to interact with this pathway^8^,^35^. Therefore, none of these functions seems to be suited to explain the phenotype of Vp447_∆c_ in cells with an activated IFN-system.

The possibility to partially overcome the IFN-induced antiviral state in cells by the incorporation of core protein into virus particles of Vp447_∆c_ indicates a so far unknown function of core protein. The differences in core integration correlating with differing infectivity of Vp447_∆c_ and Vp447_ΔcN2177_ also imply a certain ‘dose dependence’. Seemingly, the more core protein present in the virus particle, the better (at least in the case of infection of IFN-primed cells). This is also supported by the finding that the infectivity of Vp447 grown in core-expressing cells always exceeded the one of Vp447 grown in control cells. This might also provide evidence that the pestiviral nucleocapsid is not a strictly organized icosahedral structure consisting of a defined number of capsid proteins, but rather a polymorphous aggregate of viral genome and core protein. This is supported by single particle cryo electron microscopic reconstructions on other members of the *Flaviviridae*^25^,^26^,^37^. Yet, as reconstructions in cryo electron microcopy employing single particle analysis usually treat the full capsid, it is possible that a higher order nucleocapsid exists which features independent symmetric properties at a smaller scale (e.g. not the full genome).

Capsid proteins are considered one of the most essential components of virus particles^38^. Main function of the nucleocapsid is the condensation of the genome and thus it is a prerequisite for virus morphogenesis. RNA chaperone as well as RNA-binding activity have been reported for the core protein of BVDV^39^,^40^. Yet, the reported lack of a capsid coding region in some members of the genus *Pegivirus*^27^ implies compensatory mechanisms of genome packaging. Analysis of CSFV NS3 encoding for the N2177Y mutation did not reveal changes in structure or RNA helicase activity indicative of adaptation or change of function^41^. Therefore, the means of core protein free particle assembly remain unclear.

After entry into the host cell the nucleocapsid supposedly deteriorates, facilitating the early steps of replication. In terms of a plus-stranded RNA virus the initial step of replication is translation at a ribosome which requires an uncondensed and accessible RNA molecule. Devoid of its usual protein cover, it is easy to imagine the hostile environment a single RNA-genome faces when getting released into a cell with all its RNases and RNA-binding proteins. Upon activation of the IFN-system, the situation gets even more precarious. Therefore, the likelihood of a ‘naked’ viral RNA genome to find a ribosome and to stay intact long enough to allow minus-strand synthesis seems low. This is also supported by the high amounts of viral genomes (>1 × 10^5^) needed to transfect a single cell. Yet, this might also be at least partially due to defects/degradation of the *in vitro* generated transcripts and low transfection efficiency.

The growing insight into the function of IFN-stimulated genes presents several effectors core protein might protect against. RNases like RNaseL and ISG20 as well as inhibitors of protein synthesis like IFIT proteins seem the most likely candidates core protein could protect against (reviewed in ref. 4). Yet, other options remain. A differentially packaged RNA genome, in which core protein is potentially replaced by another protein, might render the virus more susceptible to changes in membrane properties as invoked by IFITM proteins (reviewed in ref. 42) or it might be more prone to interactions with capsid degrading factors like MX1 (reviewed in ref. 31).

Preliminary efforts to detect involved IFN-induced factors by transcriptome analysis using microarrays of untreated and IFN-treated SK6- and PK15-cells did not identify potential candidates. Also, overexpression of single PK15-derived IFN-induced candidate genes like OAS, RNaseL, PKR and ISG20 in SK6-cells – with or without IFN-treatment - was not sufficient to restore the phenotype observed in PK15-cells after IFN-treatment. ISGs often act in a synergistic manner^43^ (reviewed in ref. 4). Therefore, it might well be that several molecules are responsible for the inhibitory effect observed. Further research, involving ultrastructural analysis of Vp447 and Vp447_∆c_ particles and RNA seq approaches will hopefully help to shed light on mechanisms and factors contributing to the observed phenotype.

In summary, CSFV core protein could be identified as a factor important in the infection of IFN-primed cells, revealing a novel function of an integral structural component of a virus particle as an antagonist of the IFN-system.

## Materials and Methods

### Cell culture, virus rescue and virus quantification

All cells employed in this study, SK6-, PK15- and STE-cells, as wells as primary porcine cells were grown in Dulbecco’s modified Eagle’s medium supplemented with 10% BVDV-free FCS at 37 °C and 5%CO_2_. Virus was rescued from cDNA *in vitro* transcribed into RNA (SP6 polymerase, New England Biolabs), which subsequently was transfected in 5 × 10^6^ SK6-cells by electroporation (Bio-Rad Gene Pulser). Transfection efficiency was assessed 14 h after transfection by immunohistochemistry specifically staining CSFV E2 with the mouse monoclonal antibody A18. Virus containing supernatant for infection experiments was harvested 36 h after electroporation, clarified by centrifugation for 5 min at 3000 × g and stored in aliquots at −70 °C.

Titer was determined in ffu/ml on SK6-cells employing 10-fold dilution steps. 14 h after infection, cells were stained by immunohistochemistry as described above and foci of infected cells were counted using a Nikon Eclipse TS100 microscope.

### Generation of ppEndo and ppMacro cells

PpEndo were harvested from aortas of ~6 months old feeder pigs obtained at the local slaughter house. Therefore, aortas were freed of all adjacent tissue, washed several times with PBS and the intimal layer of the aorta was then scraped off with a scalpel blade. Cells were immediately seeded on tissue culture plates and used for experiments as soon as cells had grown to confluency.

PpMacro were isolated from buffy coats derived from EDTA blood of ~6 months old feeder pigs. Peripheral blood mononuclear cells were separated from other blood components by gradient centrifugation employing Ficoll Paque (GE Healthcare) (1200 g 45 min). After 2 additional wash steps with PBS, 5 × 10^6^ cells were seeded in teflon bags to allow for the differentiation into promacrophages. After 7d, cells were seeded on tissue culture plates and employed for infection experiments.

### Determination of virus growth

1 × 10^5^ SK6-, PK15-, STE-cells, ppEndo or ppMacro were seeded in 24 well plates. If applicable, cells were treated with 300 IU human IFN-α2A (Roferon, Roche) 16 h prior to or 4 h after infection. Cells were infected with a MOI of 1 for 4 h. Thereafter, medium was exchanged and virus titer was determined 24 h and 48 h after infection as described above.

### Determination of cellular susceptibility

1 × 10^5^ SK6- or PK15-cells or ppEndo were seeded in 24 well plates. Porcine cell lines were routinely treated with 300 IU human IFNα2A (Roferon, Roche) 16 h prior to infection (if not indicated otherwise). Ten-fold dilutions of the viruses to be tested were used for infection. 4 h after infection, the medium was exchanged to a medium containing 0.5% methylcellulose. 24 h after infection, cells were stained by immunohistochemistry and infected foci were counted. The permissiveness was calculated in percent employing the following formula:


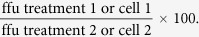


### RNA infectivity

Equal dilutions (10^−2^, 10^−3^, 10^−4^, 10^−5^) of 2.5 μg *in vitro* transcribed viral genome were transfected in SK-6 or PK15-cells resuspended in PBS by electroporation (SK6-cells: 2 mm gap, 0.180 kV, 950 μF, ∞ Ω; PK15: 4 mm gap, 0.25 kV, 950 μF, ∞ Ω). IFN-treatment was performed 16 h prior to transfection. After transfection, cells were seeded on 6 well plates and the medium was exchanged to medium containing 0.5% methylcellulose 4 h after transfection. 24 h after transfection, cells were stained by immunohistochemistry and the number of infected foci per genome dilution was determined.

### Generation of core transcomplemented viruses

*In vitro* transcribed viral genome was transfected in SK6 tet on cells expressing core 255, core-E^rns^ or core-YFP after induction with Doxycycline 16 h earlier. Virus containing supernatant was harvested 24 h after transfection and clarified at 3000× g for 5 min. Thereafter, virus was either frozen at −70 °C or pelleted by ultracentrifugation in a Beckmann Optima ultracentrifuge with a TLA45 rotor at 45000 rpm for 1 h. Pelleted virus was subsequently used for Western blot analysis.

### Western blot analysis

For the detection of E2 and core protein in concentrated virus particles, virions were resuspended in 1% SDS protein loading buffer and subjected to Western blot analysis as described in ref. 28. MX1 and beta-actin were detected in cell lysates by mouse monoclonal antibodies (MX1: M143, University of Freiburg, Germany; β-actin: A5441, Sigma-Aldrich).

### Generation of core expressing PK15-cells

For the generation of PK15-cell lines stably expressing core 255, core 267, core-YFP or YFP, HIV pseudoparticles, pseudotyped with VSV G-protein and carrying the respective coding regions, were generated in HEK 293 T cells as described in ref. 44. 1 × 10^5^ low passage PK15-cells were seeded in each well of a 6 well plate and infected with the respective pseudotype. Positive clones were selected by treatment with 1 μg/ml puromycine. Core integration and core expression were confirmed by PCR and Western blot analysis.

## Additional Information

**How to cite this article**: Riedel, C. *et al*. The core protein of a pestivirus protects the incoming virus against IFN-induced effectors. *Sci. Rep.* **7**, 44459; doi: 10.1038/srep44459 (2017).

**Publisher's note:** Springer Nature remains neutral with regard to jurisdictional claims in published maps and institutional affiliations.
